# A Comparative Study of Micromechanical Analysis Models for Determining the Effective Properties of Out-of-Autoclave Carbon Fiber–Epoxy Composites

**DOI:** 10.3390/polym16081094

**Published:** 2024-04-14

**Authors:** Young Cheol Kim, Hong-Kyu Jang, Geunsu Joo, Ji Hoon Kim

**Affiliations:** 1 Department of Composite Structure & System, Korea Institute of Materials Science (KIMS), 797, Changwon-daero, Seongsan-gu, Changwon-si 51508, Republic of Korea; yckim@kims.re.kr (Y.C.K.); hongkyu@kims.re.kr (H.-K.J.); gsjoo@kims.re.kr (G.J.); 2 Department of Mechanical Engineering, Pusan National University, Pusan 46241, Republic of Korea

**Keywords:** micromechanics models, carbon-fiber-reinforced plastic (CFRP), representative volume element (RVE), effective properties

## Abstract

This study aims to critically assess different micromechanical analysis models applied to carbon-fiber-reinforced plastic (CFRP) composites, employing micromechanics-based homogenization to accurately predict their effective properties. The paper begins with the simplest Voigt and Reuss models and progresses to more sophisticated micromechanics-based models, including the Mori–Tanaka and Method of Cells (MOC) models. It provides a critical review of the areas in which these micromechanics-based models are effective and analyses of their limitations. The numerical analysis results were confirmed through finite element simulations of the periodic representative volume element (RVE). Furthermore, the effective properties predicted by these micromechanics-based models were validated via experiments conducted on IM7/5320-1 composite material with a fiber volume fraction of 0.62.

## 1. Introduction

At present, modeling and simulation methods are widely recognized as being essential for predicting the performance of composite materials in various industrial fields, such as the automotive and aerospace industries, where they offer a cost-effective and efficient alternative to traditional experimental methods. Multiscale modeling is a method commonly employed in structural simulation, particularly for the analysis of inhomogeneous materials such as carbon-fiber-reinforced plastics (CFRPs) [1]. It can be used to predict material behavior at the macroscopic level in composite structures. In this approach, micromechanical homogenization techniques are employed to determine the effective properties of the composites, spanning various scale levels from individual fibers and inclusions to entire components under stress. Such a strategy is important for optimizing structural design, and there is a focus on developing homogenization methods and micromechanical models for CFRP. Composite material design involves defining equivalent effective macromechanical properties based on information about the microconstituents, which can be obtained using analytical, numerical, or experimental approaches.

There are several analytical micromechanical models available for predicting the effective elastic properties of composites. The micromechanics models established by Voigt [2] and Reuss [3] provide the boundaries for estimating effective properties. Additionally, there is the model of Mori–Tanaka [4], whose research focused on calculating the average internal stress in a matrix of a material containing inclusions by using eigenstrains. Their work served as the basis for a series of studies on determining the effective properties of composites, such as those by Taya and Arsenault [5]. The eigenstrain method has become a popular approach due to the simplicity of its implementation. In [6], Benveniste reformulated the Mori–Tanaka approach by introducing Eshelby’s equivalent inclusion method, which subsequently led to the development of the Mori–Tanaka tensor. This tensor enables the computation of effective properties of a composite based on Eshelby’s tensor, and this method has been widely accepted in composite research. Several studies have investigated composites containing inclusions of different shapes and sizes, such as penny-shaped and spherical forms as well as noncylindrical [7,8,9,10,11,12,13] and nonelliptical shapes [14,15,16]. Various inclusion alignment configurations, ranging from random to partially aligned distributions, have been investigated using the Eshelby–Mori–Tanaka model [17,18,19,20]. This method has also been leveraged for investigations into multiphase interactions in composites with coated fiber reinforcements [21,22] and hierarchical multi-interface models [23,24]. Although the model has been successful in application, some limitations have been identified. For example, the model cannot predict the auxetic behavior in composites with nonauxetic inclusions due to the lack of joints between inclusions [25]. The aim of recent modifications by Azoti et al. [26] to Eshelby’s tensor and the Mori–Tanaka approach was to enhance the efficacy of predicting effective properties in composites infused with graphene platelets. The Method of Cells (MOC) [27] provides a framework for conducting micromechanical analyses of composite materials, with particular emphasis on the periodic arrangement of their microstructures. This approach is particularly useful for composites where reinforcing inclusions, such as fibers, are periodically distributed. The analysis is simplified by the MOC because it focuses on a single repeating unit cell (RUC), which is essentially the basic element of the composite. The method involves applying displacement and tensile continuity conditions at interfaces within and between cells while also satisfying equilibrium conditions. This approach can be utilized to calculate the effective stiffness tensor for linear elastic composites. It is worth noting that the MOC can be modified for composites with nonlinear properties, including those that are susceptible to damage or inelastic responses, which gives it an edge over other methods [28,29,30].

Several numerical analysis techniques have been employed to estimate the elastic properties, stress, and strain in composites, including finite difference methods, boundary element analysis, and finite element analysis (FEA) [31,32,33,34]. FEA has been particularly effective in predicting the behavior of composites under different loads, as demonstrated by numerous studies. The accuracy of finite element analysis (FEA) studies can be improved by correctly applying periodic boundary conditions (PBCs). In order to predict the overall behavior of a composite material, it is necessary to create a representative volume element (RVE) and apply PBCs, as demonstrated in previous studies [32,35,36,37,38,39,40,41]. This process can be utilized to model complex scenarios, including those with discontinuous fibers and microscale voids, using a configuration of periodic RVEs whose essential parameters are randomly distributed. While numerical methods are highly accurate in capturing detailed responses, they can be limited by their requirement for significant computational and human resources. However, there are simpler and less demanding techniques that may be more appropriate. Such techniques can still provide valuable insights and are worth considering.

This study presents a comprehensive comparative study of several major micromechanical models against an entire range of fiber volume fractions, extending the scope of model applicability and validation for unidirectional CFRP fabricated by an out-of-autoclave (OOA) process. The strengths and weaknesses of these models have been assessed by comparing their results with available experimental data. The study examines four closed-form micromechanical models that have been documented in scholarly publications. Specifically, the Mori–Tanaka method extends Eshelby’s method for an inclusion in an infinite matrix to composites with interacting inclusions. MOC is an advanced method that models the composite’s microstructure as a periodic array of unit cells. It is especially useful for predicting the effective behavior of composites with a significant volume fraction of inclusions. Additionally, the FE-based RVE homogenization method is employed to assess the effective properties of composites with complex microstructures, utilizing PBCs. It is worth noting that the performance of micromechanical models can be comparatively evaluated using micromechanics-based models along with experimental studies on carbon-fiber-reinforced polymer matrix composites, providing a robust validation framework.

## 2. Analytical Microscale Approaches

Over the past decades, analytical multiscale approaches have often been reliant on micromechanical models that homogenize physically inhomogeneous materials in providing averaged mechanical properties representing equivalent behavior. In the context of analytical multiscale approaches, this process is commonly referred to as mean-field homogenization (MFH). The effective elastic properties of the homogenized composite materials can be determined by averaging mechanical field variables, such as strain and stress, in the RVE. In this section, the basic concepts and theories of analytical micromechanics-based models for CFRP composites are discussed, beginning with the basic Voigt and Reuss models and progressing to more complex approaches such as the Mori–Tanaka model and the MOC.

### 2.1. Mixing Rules: Voigt and Reuss Approximations

The simplest models are based on mixing rules that calculate the stiffness or strain of the composites from the fiber volume fraction and the stiffness of the fiber and matrix. The approaches of Voigt and Reuss should be mentioned here [2,3]:

(1) $$C_{Voigt}^{*}=C^{\left(m\right)}+V_{f}\left(C^{\left(f\right)}-C^{\left(m\right)}\right)=V_{f}C^{\left(f\right)}+V_{m}C^{\left(m\right)}$$

(2) $$S_{Reuss}^{*}=S^{\left(m\right)}+V_{f}\left(S^{\left(f\right)}-S^{\left(m\right)}\right)=V_{f}S^{\left(f\right)}+V_{m}S^{\left(m\right)}$$

where $C^{*}$ is the effective stiffness tensor and $S^{*}={\left[C^{*}\right]}^{-1}$ is the effective compliance tensor, with the fiber and matrix labeled f and m. Correspondingly, $V_{f}$ is the fiber volume fraction and $V_{m}=\left(1-V_{f}\right)$ is matrix volume fraction. In these approaches, Voigt assumes that the fiber and matrix in the RVE experience uniform strain, whereas Reuss assumes uniform stress. Therefore, the stiffness of the composite is calculated in the approach of Voigt, while the strain is calculated for Reuss. The Reuss approach is also known as the inverse mixing rule. Since both models do not include any information about the fiber geometry, they are only rough approximations for geometrically oriented (nonspherical) inclusions. In terms of determining the effective properties of the composite, the Voigt approach represents an upper limit while the Reuss approach is a lower limit.

### 2.2. Mori–Tanaka Approach

The solution for an inclusion within an infinite matrix, subjected to uniform deformation at large distances, is termed the dilute case. This foundational solution underpins various classical micromechanics theories, especially in the case of the Mori–Tanaka method. Specifically for ellipsoidal inclusions, the Eshelby [42,43] equivalent inclusion method is instrumental for determining the inclusion’s elastic fields. Eshelby’s equivalent inclusion model for the dilute case is summarized in Appendix A.

The Mori–Tanaka approach provides a framework for estimating the internal stress within a matrix that incorporates inclusions by using eigenstrains. Benveniste [6] reformulated this approach, elucidating the assumptions made within the theory. Their method employs the Mori–Tanaka tensor to relate the stresses and strains in the matrix and fiber, which are linked through a concentration tensor. The effective stiffness tensor of the composite is then derived from the fiber and matrix stiffness tensors by incorporating volume fractions. The model presumes a dilute distribution of ellipsoidal inclusions within an infinite elastic matrix, applying Eshelby’s concept [42,43] of an equivalent inclusion model to determine the strain concentration tensor. This enables the calculation of the composite’s overall stiffness, factoring in the elastic properties of both fiber and matrix.

The identity matrix, **I**, can be expressed as

(3) $$I=V_{f}A_{f}+V_{m}A_{m}$$

In the above equation, $A_{f}$ and $A_{m}$ represent the strain concentration tensors for the fiber and matrix, respectively. It should be noted that the sum of $V_{f}$ and $V_{m}$ is equal to 1 ($V_{f}+V_{m}=1$).

Using Equation (3),

(4) $$A_{m}=\frac{1}{V_{m}}\left(I-V_{f}A_{f}\right)$$

The effective stiffness of the composite, $C^{*}$, is

(5) $$C^{*}=V_{f}C^{\left(f\right)}A_{f}+V_{m}C^{\left(m\right)}A_{m}$$

Substituting Equation (4) into Equation (5) gives

(6) $$C^{*}=V_{f}C^{\left(f\right)}A_{f}+C^{\left(m\right)}\left(I-V_{f}A_{f}\right)$$

which can be expressed as

(7) $$C^{*}=C^{\left(m\right)}+V_{f}\left(C^{\left(f\right)}-C^{\left(m\right)}\right)A_{f}$$

It can similarly be shown that

(8) $$S^{*}=S^{\left(m\right)}+V_{f}\left(S^{\left(f\right)}-S^{\left(m\right)}\right)B_{f}$$

where the concentration tensors $A_{f}$ and $B_{f}$ are such that

(9) $$\begin{array}{c} {\bar{\varepsilon }}^{\left(f\right)}=A_{f}\varepsilon^{0}\\ {\bar{\sigma }}^{\left(f\right)}=B_{f}\sigma^{0} \end{array}$$

**Figure 1:**
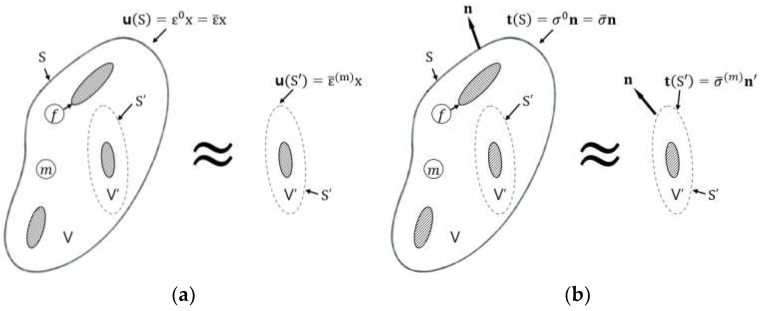
Schematic illustrations for boundary conditions of the Mori–Tanaka approach: (**a**) displacement; (**b**) traction.

For a dilute solution case, the homogenized strain ${\bar{\varepsilon }}^{\left(m\right)}$ and stress ${\bar{\sigma }}^{\left(m\right)}$ in the matrix material can be approximated using the externally applied homogeneous boundary conditions at the surface S, $\varepsilon^{0}$ and $\sigma^{0}$, respectively (Figure 1). The phase f and m represent the fiber (or inclusion) and matrix. This approximation is valid under the assumption that the presence of inclusions has a negligible effect on the overall strain and stress distribution within the matrix. Mathematically, this is represented as

(10) $${\bar{\varepsilon }}^{\left(m\right)}\approx \varepsilon^{0}=\bar{\varepsilon }\;(\mathrm{Dilute})$$

(11) $${\bar{\sigma }}^{\left(m\right)}\approx \sigma^{0}=\bar{\sigma }(Dilute)$$

The Mori–Tanaka method further examines the effect of a single inclusion within a large volume $V^{\prime }$, which is enclosed by a surface $S^{\prime }$. The boundary conditions applied to this volume dictate that the displacement field $u\left(S^{\prime }\right)$ is proportional to the homogenized strain in the matrix, scaled by the position vector x:

(12) $$u\left(S^{\prime }\right)={\bar{\varepsilon }}^{\left(m\right)}x$$

The strain within the inclusion ${\bar{\varepsilon }}^{\left(f\right)}$ is related to the matrix strain through the strain concentration tensor T, which accounts for the discrepancy in mechanical properties between the inclusion and the matrix. This tensor is crucial for predicting the behavior of the inclusion under applied strains:

(13) $${\bar{\varepsilon }}^{\left(f\right)}=T{\bar{\varepsilon }}^{\left(m\right)}$$

The overall homogenized strain $\bar{\varepsilon }$ in the composite can then be expressed as a volume-fraction-weighted sum of the matrix and fiber (inclusion) strains. This representation acknowledges that the composite’s response to external loading is a composite effect of both its constituents. From Equation (10), $\bar{\varepsilon }$ can be expressed as

(14) $$\bar{\varepsilon }=V_{m}{\bar{\varepsilon }}^{\left(m\right)}+V_{f}{\bar{\varepsilon }}^{\left(f\right)}=\varepsilon^{0}$$

Substituting Equation (13) into Equation (14) and then rewriting yields

(15) $${\bar{\varepsilon }}^{\left(m\right)}={\left(V_{m}I+V_{f}T\right)}^{-1}\varepsilon^{0}$$

By substituting Equation (15) into Equation (13) again and associating the result with the first of Equation (9), one can obtain

(16) $${\bar{\varepsilon }}^{\left(f\right)}=T{\left(V_{m}I+V_{f}T\right)}^{-1}\varepsilon^{0}$$

Comparing Equation (16) with Equation (9), it can be seen that the strain concentration tensor, T, for the Mori–Tanaka method can be expressed by

(17) $$A_{f}=T{\left(V_{m}I+V_{f}T\right)}^{-1}$$

From Equation (13) and the Mori–Tanaka premise, it can be inferred that the mean matrix strain reflects the applied strain in the dilute scenario. This implies that T corresponds to Eshelby’s solution for strain concentration, specifically $T=A_{f}^{\mathrm{dilute}\;}$. As a result, the strain concentration tensor of the Mori–Tanaka method, $A_{f}^{MT}$, can be expressed in terms of $A_{f}^{\mathrm{dilute}\;}$:

(18) $$A_{f}^{MT}=A_{f}^{\mathrm{dilute}\;}{\left[V_{m}I+V_{f}A_{f}^{\mathrm{dilute}\;}\right]}^{-1}$$

Finally, by substituting Equation (18) into Equation (7), one can obtain the effective stiffness of the composite.

(19) $$C^{*}=C^{\left(m\right)}+V_{f}\left(C^{\left(f\right)}-C^{\left(m\right)}\right)A_{f}^{\mathrm{dilute}\;}{\left(V_{m}I+V_{f}A_{f}^{\mathrm{dilute}\;}\right)}^{-1}$$

### 2.3. The Method of Cells

**Figure 2:**
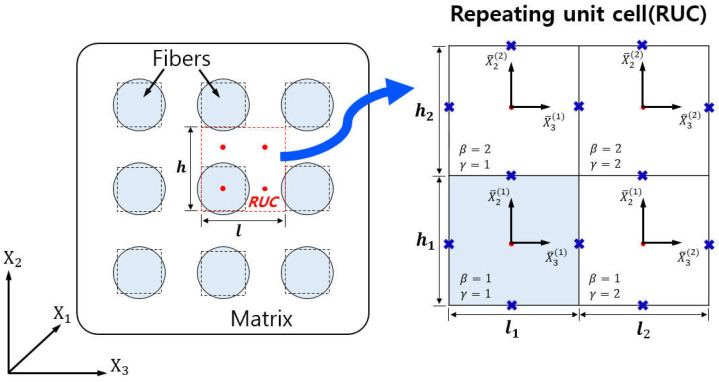
Simplified components of composite with periodic array fibers and 2 × 2 architecture of the MOC.

The micromechanics-based MOC model is dependent on the periodicity in the microstructure of composite materials. Specifically, it focuses on the regular arrangement of reinforcing fibers [27]. This technique allows studying a single RUC instead of the entire composite. It focuses on displacement and traction continuity, as well as equilibrium, at both intra- and inter-cell interfaces. Compared to previous methods, the MOC has a wider range of applicability due to its ability to handle nonlinear behaviors, such as damage or inelasticity, in composite constituents [29,30]. Using the model, a composite with continuously reinforced fibers is considered as a doubly periodic array, which implies infinite repetition in two directions. The RUC of the MOC consists of four subcells, which are identified by centroids (indicated by red dots) representing one fiber and three matrix points in Figure 2. These centroids indicate regions of influence rather than precise shapes. It is important to note that the MOC eliminates corner stress risers by portraying fibers as pseudo-circular for analytical purposes.

Using the MOC framework, the cross-sectional area of a fiber in a composite is defined as $h_{1}l_{1}$, while $h_{2}$ and $l_{2}$ indicate the fiber spacing within the matrix. This structure enables the analysis of an RUC as depicted in Figure 2, which comprises four subcells identified by β,γ=1,2. In order to model this, four local coordinate systems $\left(x_{1},{\bar{x}}_{2}^{\left(\beta \right)},{\bar{x}}_{3}^{\left(\gamma \right)}\right)$ are established, each centered at a subcell’s centroid. To achieve balanced composite behavior, linear displacement expansion is applied relative to subcell center distances. This approach utilizes first-order theory, with the displacement within each subcell then being expressed as follows:

(20) $$u_{i}^{\left(\beta \gamma \right)}=w_{i}^{\left(\beta \gamma \right)}\left(x\right)+{\bar{x}}_{2}^{\left(\beta \right)}\varphi_{i}^{\left(\beta \gamma \right)}+{\bar{x}}_{3}^{\left(\gamma \right)}\psi_{i}^{\left(\beta \gamma \right)}\;i=1,2,3$$

where $w_{i}^{\left(\beta \gamma \right)}(x)$ represents the displacements located at the center of subcell, and $\varphi_{i}^{\left(\beta \gamma \right)}$ and $\psi_{i}^{\left(\beta \gamma \right)}$ denote the linear dependence of the displacements on local coordinates. The displacement gradients are then correlated with strain components of subcells, employing standard strain–displacement relationships:

(21) $$\varepsilon_{ij}^{\left(\beta \gamma \right)}=\frac{1}{2}\left[\partial_{j}u_{i}^{\left(\beta \gamma \right)}+\partial_{i}u_{j}^{\left(\beta \gamma \right)}\right]\;i,j=1,2,3$$

where $\partial_{1}=\partial /\partial x_{1},\partial_{2}=\partial /\partial {\bar{x}}_{2}^{\left(\beta \right)}$, and $\partial_{3}=\partial /\partial {\bar{x}}_{3}^{\left(\gamma \right)}$

By substituting Equation (21) into Equation (20), the strain field for a subcell is generated. The resulting strain values are

(22) $$\begin{array}{c} \varepsilon_{11}^{\left(\beta \gamma \right)}=\frac{\partial }{\partial x_{1}}w_{1}\\ \varepsilon_{22}^{\left(\beta \gamma \right)}=\phi_{2}^{\left(\beta \gamma \right)},\\ \varepsilon_{33}^{\left(\beta \gamma \right)}=\psi_{3}^{\left(\beta \gamma \right)},\\ 2\varepsilon_{23}^{\left(\beta \gamma \right)}=\phi_{3}^{\left(\beta \gamma \right)}+\psi_{2}^{\left(\beta \gamma \right)},\\ 2\varepsilon_{13}^{\left(\beta \gamma \right)}=\psi_{1}^{\left(\beta \gamma \right)}+\frac{\partial }{\partial x_{1}}w_{3},\\ 2\varepsilon_{12}^{\left(\beta \gamma \right)}=\phi_{1}^{\left(\beta \gamma \right)}+\frac{\partial }{\partial x_{1}}w_{2} \end{array}$$

In the case of orthotropic materials, subcell stresses are expressed in terms of their corresponding strains by using Hooke’s law. The equation is written as follows:

(23) $$\begin{array}{cc} \left[\begin{array}{c} \sigma_{11}^{\left(\beta \gamma \right)}\\ \sigma_{22}^{\left(\beta \gamma \right)}\\ \sigma_{33}^{\left(\beta \gamma \right)}\\ \sigma_{23}^{\left(\beta \gamma \right)}\\ \sigma_{13}^{\left(\beta \gamma \right)}\\ \sigma_{12}^{\left(\beta \gamma \right)} \end{array}\right]=\left[\begin{array}{cccccc} C_{11}^{\left(\beta \gamma \right)} & C_{12}^{\left(\beta \gamma \right)} & C_{13}^{\left(\beta \gamma \right)} & 0 & 0 & 0\\ C_{12}^{\left(\beta \gamma \right)} & C_{22}^{\left(\beta \gamma \right)} & C_{23}^{\left(\beta \gamma \right)} & 0 & 0 & 0\\ C_{13}^{\left(\beta \gamma \right)} & C_{23}^{\left(\beta \gamma \right)} & C_{33}^{\left(\beta \gamma \right)} & 0 & 0 & 0\\ 0 & 0 & 0 & C_{44}^{\left(\beta \gamma \right)} & 0 & 0\\ 0 & 0 & 0 & 0 & C_{55}^{\left(\beta \gamma \right)} & 0\\ 0 & 0 & 0 & 0 & 0 & C_{66}^{\left(\beta \gamma \right)} \end{array}\right]\left[\begin{array}{c} \varepsilon_{11}^{\left(\beta \gamma \right)}\\ \varepsilon_{22}^{\left(\beta \gamma \right)}\\ \varepsilon_{33}^{\left(\beta \gamma \right)}\\ 2\varepsilon_{23}^{\left(\beta \gamma \right)}\\ 2\varepsilon_{13}^{\left(\beta \gamma \right)}\\ 2\varepsilon_{12}^{\left(\beta \gamma \right)} \end{array}\right] & \end{array}$$

For constituents that exhibit transverse isotropy with 2–3 isotropy, the following relationships hold: $C_{33}^{\left(\beta \gamma \right)}=C_{22}^{\left(\beta \gamma \right)},C_{13}^{\left(\beta \gamma \right)}=C_{12}^{\left(\beta \gamma \right)},C_{55}^{\left(\beta \gamma \right)}=C_{66}^{\left(\beta \gamma \right)}$, and $C_{44}^{\left(\beta \gamma \right)}=\frac{1}{2}\left(C_{22}^{\left(\beta \gamma \right)}-C_{23}^{\left(\beta \gamma \right)}\right)$.

The effective stiffness tensor, $C^{*}$, can be expressed as follows:

(24) $$C^{*}=\frac{1}{\left(h_{1}+h_{2}\right)\left(l_{1}+l_{2}\right)}\sum_{\beta ,\gamma =1}^{2}\;h_{\beta }l_{\gamma }C^{\left(\beta \gamma \right)}A^{\left(\beta \gamma \right)}$$

where $A^{\left(\beta \gamma \right)}$ denotes strain concentration tensors in the 2 × 2 subcells. $h_{\beta }l_{\gamma }/\left(h_{1}+h_{2}\right)\left(l_{1}+l_{2}\right)$ is an expression representing the volume fraction of each subcell.

In cases where the RUC and a fiber have equal dimensions (i.e., $h_{1}=l_{1}$ and $h_{2}=l_{2}$), specific equalities apply to the elasticity constants: $C_{12}^{*}=C_{13}^{*},$ $C_{22}^{*}=C_{33}^{*}$, and $C_{55}^{*}=C_{66}^{*}$. This configuration results in six independent elastic constants, deviating from the five typically associated with transverse isotropy. To achieve composite-level transverse isotropy within the MOC, an averaging procedure is applied. To obtain the five effective Young’s modulus values, an integration process is used to average the strain concentration tensor $A^{\left(\beta \gamma \right)}$, taking into account the tensor’s rotation by an angle ξ around the $x_{1}$ (fiber)-direction. This method was described by Brayshaw [44] in work from which five effective Young’s modulus values resulted. The averaging procedure is as follows:

(25) $${\hat{A}}^{\left(\beta \gamma \right)}=\frac{2}{\pi }\int_{-\pi /4}^{\pi /4}A_{\xi }^{\left(\beta \gamma \right)}d\xi$$

The components of $A_{\xi }^{\left(\beta \gamma \right)}$ are given by

(26) $$A_{ijkl(\xi )}^{\left(\beta \gamma \right)}=T_{ip}T_{jq}T_{kr}T_{ls}A_{pqrs}^{\left(\beta \gamma \right)}$$

where in the coordinate transformation matrix, T, all indices run from 1 to 3. The matrix is given by

(27) $$T=\left[\begin{array}{ccc} 1 & 0 & 0\\ 0 & \cos\xi & \sin\xi \\ 0 & -\sin\xi & \cos\xi \end{array}\right]$$

The expressions that result are

(28) $$\begin{array}{c} {\hat{A}}_{11}^{\left(\beta \gamma \right)}=A_{11}^{\left(\beta \gamma \right)}\\ {\hat{A}}_{21}^{\left(\beta \gamma \right)}=\left(\frac{1}{2}+\frac{1}{\pi }\right)A_{21}^{\left(\beta \gamma \right)}+\left(\frac{1}{2}-\frac{1}{\pi }\right)A_{31}^{\left(\beta \gamma \right)},\\ {\hat{A}}_{31}^{\left(\beta \gamma \right)}=\left(\frac{1}{2}-\frac{1}{\pi }\right)A_{21}^{\left(\beta \gamma \right)}+\left(\frac{1}{2}+\frac{1}{\pi }\right)A_{31}^{\left(\beta \gamma \right)},\\ {\hat{A}}_{22}^{\left(\beta \gamma \right)}=\left(\frac{3}{8}+\frac{1}{\pi }\right)A_{22}^{\left(\beta \gamma \right)}+\left(\frac{3}{8}-\frac{1}{\pi }\right)A_{33}^{\left(\beta \gamma \right)}+\frac{1}{8}\left(A_{23}^{\left(\beta \gamma \right)}+A_{32}^{\left(\beta \gamma \right)}\right)+\frac{1}{4}A_{44}^{\left(\beta \gamma \right)},\\ {\hat{A}}_{23}^{\left(\beta \gamma \right)}=\left(\frac{3}{8}+\frac{1}{\pi }\right)A_{23}^{\left(\beta \gamma \right)}+\left(\frac{3}{8}-\frac{1}{\pi }\right)A_{32}^{\left(\beta \gamma \right)}+\frac{1}{8}\left(A_{22}^{\left(\beta \gamma \right)}+A_{33}^{\left(\beta \gamma \right)}\right)-\frac{1}{4}A_{44}^{\left(\beta \gamma \right)},\\ {\hat{A}}_{33}^{\left(\beta \gamma \right)}=\left(\frac{3}{8}+\frac{1}{\pi }\right)A_{33}^{\left(\beta \gamma \right)}+\left(\frac{3}{8}-\frac{1}{\pi }\right)A_{22}^{\left(\beta \gamma \right)}+\frac{1}{8}\left(A_{23}^{\left(\beta \gamma \right)}+A_{32}^{\left(\beta \gamma \right)}\right)+\frac{1}{4}A_{44}^{\left(\beta \gamma \right)},\\ {\hat{A}}_{44}^{\left(\beta \gamma \right)}=\frac{1}{4}\left(A_{22}^{\left(\beta \gamma \right)}+A_{33}^{\left(\beta \gamma \right)}\right)-\frac{1}{4}\left(A_{23}^{\left(\beta \gamma \right)}+A_{32}^{\left(\beta \gamma \right)}\right)+\frac{1}{2}A_{44}^{\left(\beta \gamma \right)},\\ {\hat{A}}_{55}^{\left(\beta \gamma \right)}=\left(\frac{1}{2}+\frac{1}{\pi }\right)A_{55}^{\left(\beta \gamma \right)}+\left(\frac{1}{2}-\frac{1}{\pi }\right)A_{66}^{\left(\beta \gamma \right)}\\ {\hat{A}}_{66}^{\left(\beta \gamma \right)}=\left(\frac{1}{2}-\frac{1}{\pi }\right)A_{55}^{\left(\beta \gamma \right)}+\left(\frac{1}{2}+\frac{1}{\pi }\right)A_{66}^{\left(\beta \gamma \right)} \end{array}$$

In Equation (24), the strain concentration tensor $A^{\left(\beta \gamma \right)}$ can be replaced by ${\overset{ˆ}{A}}^{\left(\beta \gamma \right)}$. Finally, the effective Young’s modulus values of a transversely isotropic unidirectional composite are determined:

(29) $$C^{*}=\frac{1}{\left(h_{1}+h_{2}\right)\left(l_{1}+l_{2}\right)}\sum_{\beta ,\gamma =1}^{2}\;h_{\beta }l_{\gamma }C^{\left(\beta \gamma \right)}{\overset{ˆ}{A}}^{\left(\beta \gamma \right)}$$

Equation (29) is expanded as

(30) $$C^{*}=\frac{h_{1}l_{1}C^{\left(11\right)}{\overset{ˆ}{A}}^{\left(11\right)}+h_{1}l_{2}C^{\left(12\right)}{\overset{ˆ}{A}}^{\left(12\right)}+h_{2}l_{1}C^{\left(21\right)}{\overset{ˆ}{A}}^{\left(21\right)}+h_{2}l_{2}C^{\left(22\right)}{\overset{ˆ}{A}}^{\left(22\right)}}{\left(h_{1}+h_{2}\right)\left(l_{1}+l_{2}\right)}$$

As stated, when the parameters satisfy $h_{1}=l_{1}$ and $h_{2}=l_{2}$, Equation (30) provides effective stiffness tensor components resulting in five independent elastic constants and transversely isotropic behavior, $C_{12}^{*}=C_{13}^{*}$, $C_{22}^{*}=C_{33}^{*}$, $C_{55}^{*}=C_{66}^{*}$, and $C_{44}^{*}=\frac{1}{2}\left(C_{22}^{*}-C_{23}^{*}\right)$. This function calculates the micromechanics for the Method of Cells (MOC) and produces both averaged and unaveraged results (MOCu).

## 3. Finite Element Analysis and Experiments

### 3.1. Representative Volume Element (RVE) Generation

**Figure 3:**
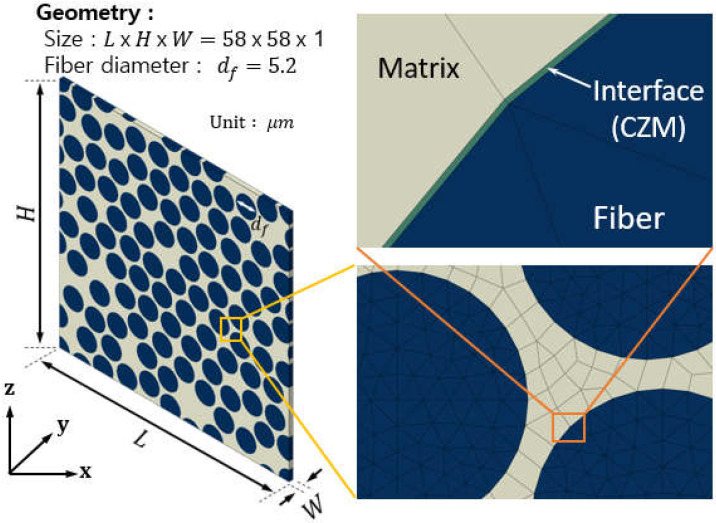
Components and geometry of the finite element model for periodic RVEs.

In computational micromechanics, FEA is employed to predict the effective properties of composite materials. The RVE model was established using Abaqus/Standard 2017 for discretization. It includes unidirectional cylindrical fibers periodically and stochastically distributed within a polymer matrix. This configuration is intended to simulate the cross-section of a unidirectional composite laminate, which is consistent with previous studies [45,46]. It is worth mentioning that in the FE simulation, the volume fraction of fiber reinforcement was set to 62%. This value was determined by measuring specimens taken from composite laminates made of CYCOM 5320-1 IM7 12K prepreg (Syensqo, Brussels, Belgium) according to the ASTM specifications for testing of mechanical properties. According to the product datasheet [47], the diameter of the carbon fiber measures 5.2 µm. To ensure computational efficiency while capturing the fundamental effective elastic properties, a square RVE with a length of 58 µm was considered sufficient [45]. The RVE with a volume fraction of 0.62 consists of 98 fibers evenly dispersed along each axis of the plane of transverse isotropy. As shown in Figure 3, a typical RVE was extruded along the axis of fibers with a thickness of w = 1.0 µm. The fibers were modeled using six-node fully integrated wedge iso-parametric elements (C3D6), while the matrix was modeled using eight-node fully integrated brick iso-parametric elements (C3D8), and eight-node cohesive iso-parametric elements (COH3D8) were used at the fiber–matrix interface. An element size of approximately 0.5 µm was chosen, assuming perfect and homogeneous contact between the fibers and matrix, with no gaps at the interface. The impact of fiber damage initiation mechanisms, such as fiber debonding and matrix shear yielding, on the effective properties was not explored in that study.

### 3.2. Periodic Boundary Conditions (PBCs)

**Figure 4:**
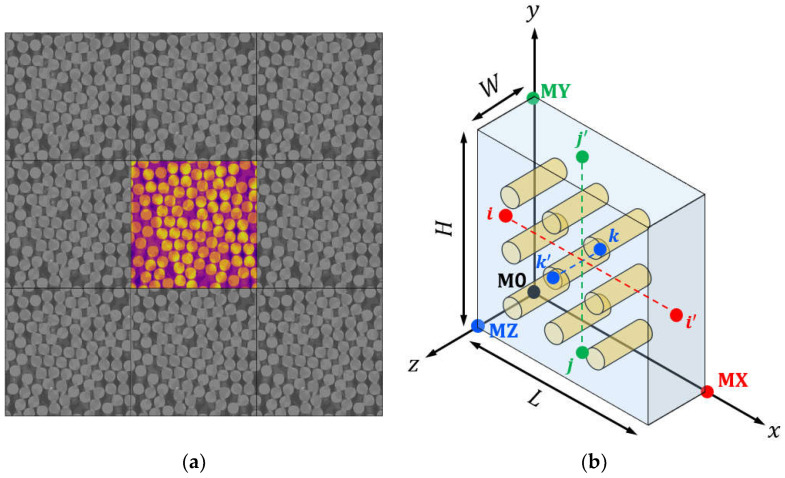
Schematic illustrations of a typical RVE under periodic boundary conditions: (**a**) periodical RVEs; (**b**) 3-dimensional; (3D) RVE model for the application of boundary conditions.

PBCs are often used to minimize the impact of boundary constraints on the mechanical behavior of the RVE model. This approach is generally preferred over iso-strain or iso-stress methods because it can more accurately replicate the mechanical response within a finite RVE size [48,49]. However, for the three types of boundary conditions, there will often be convergence to the same result if the RVE is large enough, and PBCs are often considered the optimal choice for achieving desired results in a given RVE size. In 3D RVEs, PBCs can be expressed as three displacement vectors: $u_{1},u_{2}$, and $u_{3}$. These vectors relate the relative displacement of a set of master nodes (typically M0, MX, MY, and MZ) located on opposite faces. Therefore, $u_{1}=u^{MX}-u^{M0},u_{2}=u^{MY}-u^{M0}$, and $u_{3}=u^{MZ}-u^{M0}$. In order to maintain displacement continuity with neighboring RVEs, PBCs were applied between opposite faces of the RVE, creating a pattern similar to a jigsaw puzzle, as shown in Figure 4a. The PBCs can be expressed mathematically as follows:

(31) $$\begin{array}{c} u\left(L,y,z\right)-u\left(0,y,z\right)=u^{MX}-u^{M0}=u_{1}\\ u\left(x,H,z\right)-u\left(x,0,z\right)=u^{MY}-u^{M0}=u_{2}\\ u(x,y,W)-u(x,y,0)=u^{MZ}-u^{M0}=u_{3} \end{array}$$

In the configuration shown in Figure 4b, it can be observed that the displacement between two nodes on opposite faces is equal to the displacement of the corresponding master node pair, where the direction of the master nodes and the node pairs are indicated by the colors. To allow for the application of different loading scenarios, it is suggested that displacements are applied to these master nodes. These displacements should represent any physically uniform deformation scenario that can occur within the unit cell. It should be noted that each master node aggregates the reaction forces exerted by all nodes on its associated face. For instance, to induce a uniaxial tensile strain along the x-axis, one could assign $u^{MX}=(\delta ,0,0{)}^{T},u^{MY}={\left(0,u_{y},0\right)}^{T}$, and $u^{MZ}=$ ${\left(0,0,u_{z}\right)}^{T}$. The values of $u_{y}$ and $u_{z}$ can be determined from the homogenized traction forces on their respective faces at x=L and y=H, as described in Equation (32) and in accordance with Equation (31). The representative volume element (RVE) in Abaqus utilizes the *EQUATION keyword to implement PBCs.

(32) $$\begin{array}{c} \int_{\Omega_{y}}t_{y}d\Omega =0\;\Omega_{y}:y=H\\ \int_{\Omega_{z}}t_{z}d\Omega =0\;\Omega_{z}:z=W \end{array}$$

### 3.3. Constitutive Models of Material

#### 3.3.1. Fiber

In periodic RVE analysis, carbon fibers are modeled as materials with linear elasticity and transverse isotropy. The mechanical characteristics of the IM7 carbon fiber were derived from the manufacturer’s datasheet and supplementation by previous studies [47,50,51,52]. The results are shown in Table 1. In this analysis, it was decided that nonlinear elasticity and longitudinal fractures of the fibers would be intentionally excluded from consideration.

#### 3.3.2. Epoxy Matrix

**Figure 5:**
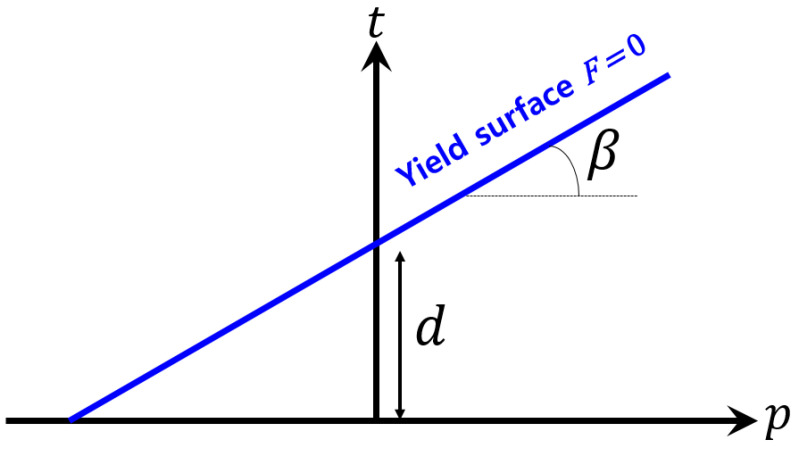
Yield surfaces in the meridional plane for linear Drucker–Prager models.

The elastoplastic behavior of polymer matrices can be significantly influenced by conditions of hydrostatic stress. In the case of epoxy polymer matrices, it has been observed that the yield behavior is pressure-dependent. This behavior can be explained by implementing the linear Drucker–Prager model in Abaqus [53]. The Drucker–Prager model is a plasticity model that describes the yield behavior of materials under pressure-dependent conditions. The model assumes that the yield surface can be represented as a circular cone in the principal stress space, with the apex located at the hydrostatic axis. The linear yield surface of this model can be expressed as follows (Figure 5):

(33) t−p⋅tanβ−d=0

where t denotes the deviatoric stress, p is the hydrostatic pressure, *β* is the friction coefficient, and d is the cohesion property. This yield surface is represented as a line in the p−t domain with a slope tanβ and intersects the vertical axis at t(p=0)=d. t is defined as

(34) $$t=\frac{1}{2}q\left[1+\frac{1}{K}-\left(1-\frac{1}{K}\right)\cdot {\left(\frac{r}{q}\right)}^{3}\right]$$

The model is defined by two parameters: the friction angle β measures the slope of the yield surface, while the dilatancy angle ψ measures its expansion. The properties of the epoxy polymer matrix used in the carbon-fiber-reinforced composites were obtained from the literature [54,55,56,57], as shown in Table 2.

#### 3.3.3. Fiber–Matrix Interface

The fiber–matrix interface was modeled utilizing the cohesive zone model, which is defined according to a mixed-mode bilinear traction–separation law [58,59,60]. There are cohesive elements at the fiber–matrix interface that are subject to a quadratic stress criterion [61]. The linear behavior ends at the onset of damage, which is dictated by a maximum stress criterion expressed as

(35) $${\left(\frac{\left\langle t_{n}\right\rangle }{N^{c}}\right)}^{2}+{\left(\frac{t_{s}}{S^{c}}\right)}^{2}+{\left(\frac{t_{t}}{S^{c}}\right)}^{2}=1$$

where ⟨⟩ denote McCaulay brackets, which are defined as ⟨x⟩=(x+|x|)/2. $t_{n}$ and $t_{s}$ represent the normal traction and shear components of the traction vector. Both shear directions, s and t, are assumed to be equal. $N^{c}$ is normal strength and $S^{c}$ is shear strength. The cohesive elements exhibit linear elastic behavior with very high penalty stiffness, $k_{nn}^{c}$ and $k_{ss}^{c}$, until the damage regime initiates at $\delta_{0}$. Linear softening is commonly utilized to represent stiffness degradation leading up to the complete failure of the material ($\delta_{u})$. The dissipation of energy under mixed-mode loading is calculated using the Benzeggagh–Kenane (BK) law [62]. The criterion is expressed as follows:

(36) $$G_{C}={G_{n}^{C}+\left(G_{s}^{C}-G_{n}^{C}\right)\left\{\frac{2G_{s}}{G_{n}+{2G}_{s}}\right\}}^{\eta_{BK}}$$

where $G_{n}^{C}$ and $G_{s}^{C}$ represent the critical fracture energy in normal and shear directions, respectively. $G_{n}$ and $G_{s}$ are the reciprocal work under mixed-mode propagation. $\eta_{BK}$ denotes the BK power exponent. Table 3 presents a summary of the parameters for FEA that are specific to the IM7/5320-1 materials. This includes the properties of the fiber–matrix interface, which have been sourced from the literature [63].

## 4. Experimental Results

### 4.1. Manufacturing Process for IM7/ 5320-1 Composites

**Figure 6:**
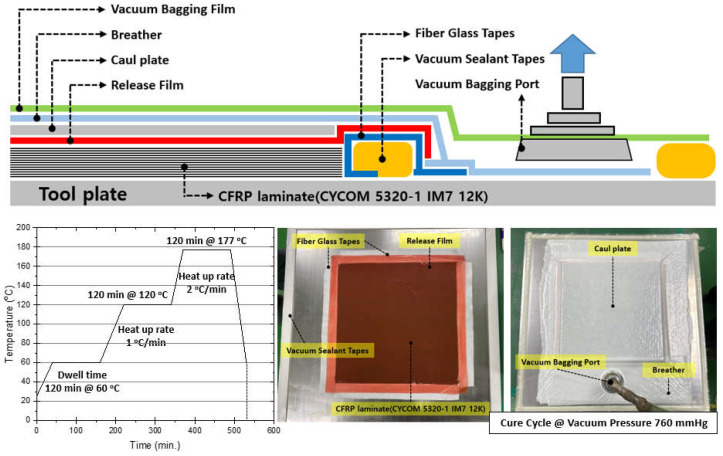
Fabrication of CYCOM 5320-1 IM7 12K VBO prepreg.

The CYCOM 5320-1 IM7 12K composite material is a prepreg system that incorporates toughened epoxy resin. It is engineered for primary structural component fabrication via vacuum-bag-only (VBO) or out-of-autoclave (OOA) processes [64]. This system is advantageous for prototyping that demands either low-cost tooling or VBO curing methods, thanks to its ability to cure at lower temperatures. CYCOM 5320-1 enables the production of autoclave-quality components with minimal porosity through vacuum-bag curing while retaining the ease of use typical of standard prepregs. This system allows the provision of mechanical properties similar to those of other toughened epoxy prepreg systems that require autoclave curing, withstanding temperatures up to 350 °F (177 °C), subject to a post-cure process at 350 °F (177 °C). Furthermore, it offers flexibility in cure cycle options [65]. Illustrated in Figure 6 is the bagging material setup with a standard VBO cure cycle and fabrication method.

### 4.2. Characterization of Unidirectional Carbon Fiber Composites

Determination of the fiber volume fraction in the carbon composite was executed using gravimetric analysis as prescribed by the ASTM standards [66,67]. Three rectangular samples, each with an area of 20 × 20 mm^2^, were cut from the composite laminate fabricated for characterization of fiber volume fraction. The density of the composite samples was measured using the buoyancy method based on Archimedes’ principle, while the fiber weight fraction of the samples was determined using the resin burn-off technique. The fiber volume fraction was calculated using formulas specified by ASTM standards, with the densities of the carbon fiber and matrix noted as 1.78 g/cm^3^ and 1.31 g/cm^3^, respectively [47,64]. The resulting fiber volume fraction was determined as 62 ± 0.6%, with a porosity of 1.9 ± 0.2%.

**Figure 7:**
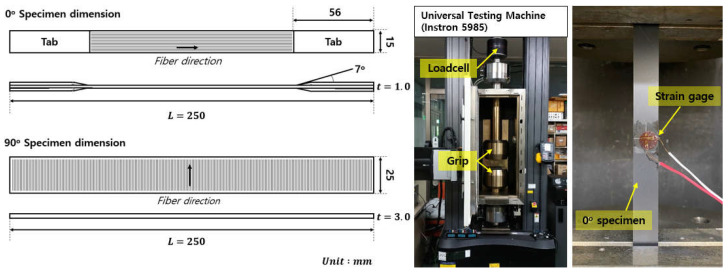
Geometry of CFRP tensile specimens according to fiber direction and experimental setup.

The CFRP plate was machined using a diamond wheel cutter (KCA, Gimhae, Korea) and the dimensions are shown in Figure 7. Tensile tests were conducted on composites in accordance with ASTM D3039/D3039M-17 standards, with five specimens per test case [68,69]. Composite material testing was performed using a Instron 5985 universal testing machine (Instron, Norwood MA, USA) equipped with a 250 kN load cell. The crosshead speed was set at a uniform 2 mm/min for all specimens. Strain was measured using 5 mm bi-axial strain gauges (Koywa, Tokyo, Japan) with a 2.1 gauge factor placed at the center of the test specimens. The properties of the in-plane shear properties for the composites were taken from previous research [70]. The mechanical test results for IM7/5320-1 composites (Vf = 62%) were compared to those of micromechanical models.

## 5. Comparison of Predicted Effective Properties for the Micromechanics Models

In this section, the results of four closed micromechanics models—Voigt, Reuss approximation, Mori–Tanaka, and MOC—are analyzed together with the results of FEA. The four analytical closed-form micromechanics models were implemented using MATLAB code (MATLAB R2023a). The effective properties of the composites were assessed through a computational homogenization scheme that utilized PBCs for each representative volume element (RVE). The simulation time of the RVE model was about 120 s for the baseline case using six cores with an Intel Core™ i5-10400 @ 2.90 GHz (Intel, Santa Clara CA, USA). The constitutive matrix was obtained by post-processing element-wise stress and volume, which were then converted to the effective elastic Young’s modulus. Equations for calculating the effective properties of unidirectional fiber-reinforced composites with transverse isotropy are described in Appendix B. Lastly, the two most significant results of the micromechanics models are analyzed as follows:

1. Four analytical micromechanical models and finite element analysis are utilized to compare the predicted effective properties of an IM7/5320-1 unidirectional CFRP with a fiber volume fraction of 0.62.
2. The effective properties of all the micromechanics models are compared for all fiber volume fractions ranging from 0.0 to 1.0.

### 5.1. Comparison of the Predicted Effective Properties of CFRP with a Fiber Volume Fraction of 62%

**Figure 8:**
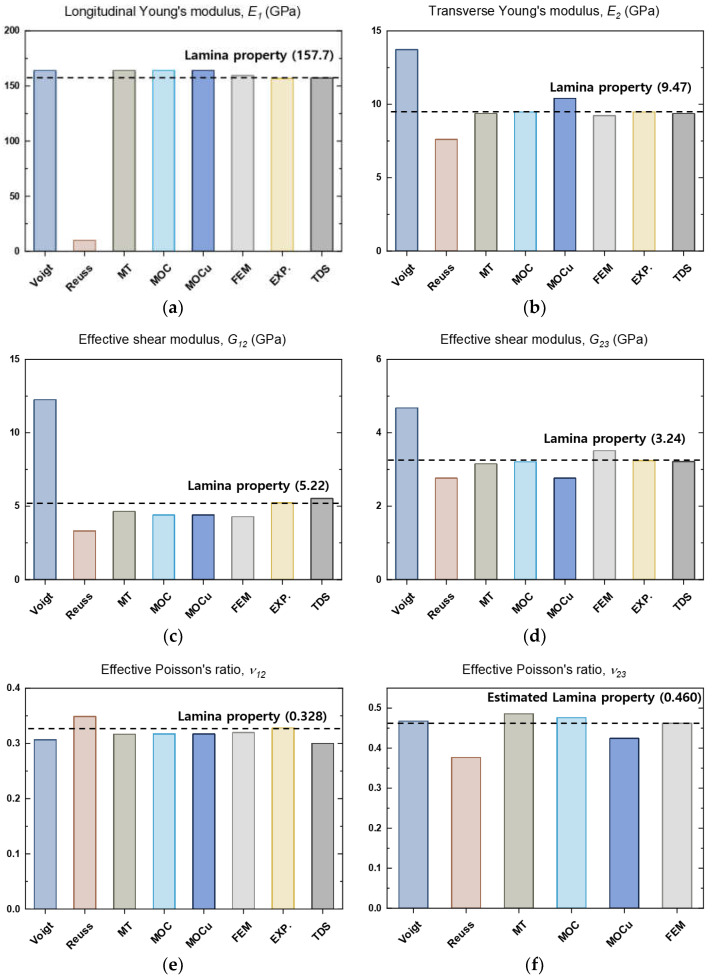
Bar charts for comparison of predicted effective properties of unidirectional IM7/5320-1 CFRP with fiber volume fraction of 0.62 by different micromechanical models: (**a**) longitudinal Young’s modulus, $E_{1}$; (**b**) transverse Young’s modulus, $E_{2}$; (**c**) shear modulus, $G_{12}$; (**d**) shear modulus, $G_{23}$; (**e**) Poisson’s ratio, $\upsilon_{12}$; (**f**) Poisson’s ratio, $\upsilon_{23}$.

In this study, a comparison of the effective properties of a unidirectional IM7/5320-1 CFRP with a fiber volume fraction of 0.62 is conducted. The results were obtained from analytical micromechanics-based models, FEA, experiments, and the company’s technical datasheet (TDS) [64], as shown in Figure 8. The results of the tests performed in Section 4.2 are compared against the lamina mechanical properties of CFRP, which are represented by the dotted horizontal line. All models, with the exception of the Reuss model, have been found to accurately predict the longitudinal modulus of elasticity. This is supported by both the finite element and experimental results. In the graph, MT stands for Mori–Tanaka. The term ‘MOC’ refers to results obtained through averaging to determine the effective properties for transverse isotropy, while unaveraged predictions are referred to as ‘MOCu’. It is clear from the results that all micromechanical models, except the Voigt and Reuss models, show relatively good agreement.

### 5.2. Comparison of the Predicted Effective Properties of CFRP as a Function of Fiber Volume Fractions

**Figure 9:**
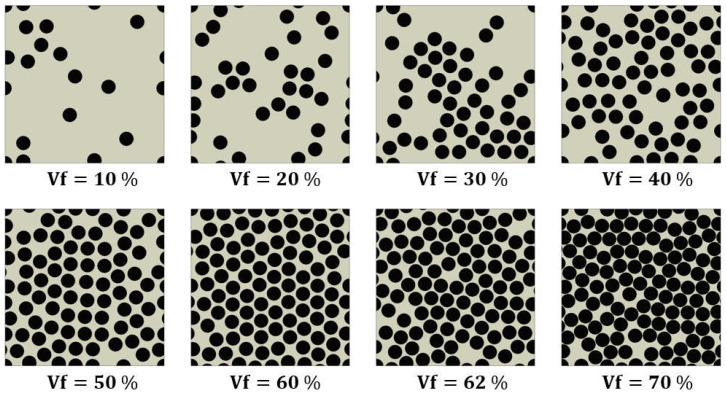
Configuration of RVE models with eight different fiber volume fractions.

This section presents a comparison of the predicted effective properties of micromechanics-based models for unidirectional IM7/5320-1 composites for the entire range of fiber volume fractions. The effective properties were analyzed using 3D RVE models generated at different volume fractions ranging from 10% to 70%, as shown in Figure 9, with 62% of the data obtained from experimental results.

**Figure 10:**
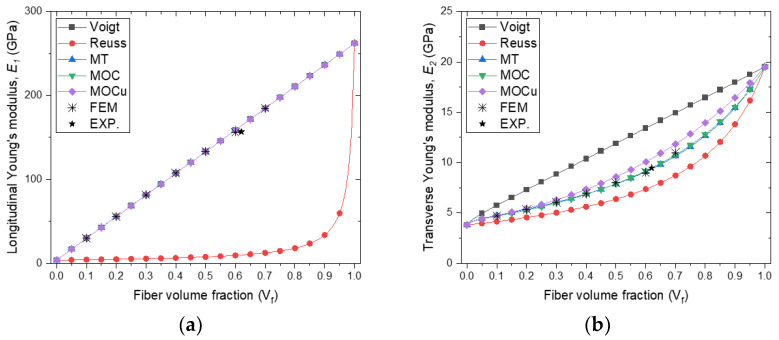

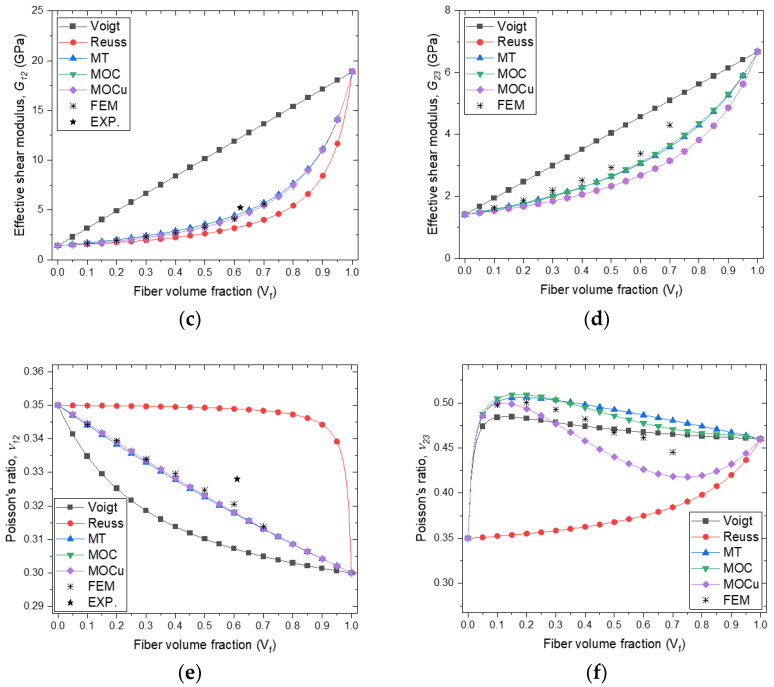
Comparison of predicted effective properties of unidirectional IM7/5320-1 CFRP as a function of volume fraction by different micromechanical models: (**a**) longitudinal Young’s modulus, $E_{1}$; (**b**) transverse Young’s modulus, $E_{2}$; (**c**) shear modulus, $G_{12}$; (**d**) shear modulus, $G_{23}$; (**e**) Poisson’s ratio, $\upsilon_{12}$; (**f**) Poisson’s ratio, $\upsilon_{23}$.

Figure 10 presents a comparison of the predicted effective properties of unidirectional IM7/5320-1 CFRP using different micromechanical models as a function of volume fraction. It is worth noting that, at a volume fraction of 0, the effective properties are equivalent to those of the matrix material, whereas at a volume fraction of 1, the effective properties are equivalent to those of the fiber material. Figure 10a,b present the longitudinal Young’s modulus and transverse Young’s modulus predicted by analytical micromechanical models and FEA. It is worth noting that the Voigt and Reuss approaches provide upper and lower limits for the composite stiffness. Additionally, all methods, except for Reuss, predict a linear relationship between the longitudinal Young’s modulus, $E_{1}$, and fiber volume fraction. The Reuss prediction of $E_{1}$ exhibits a significant deviation from the other methods in displaying a highly nonlinear relationship with volume fraction. Furthermore, there is a noticeable contrast in the plots of transverse Young’s modulus, E2, between the micromechanical models, as illustrated in Figure 10b. It is worth noting that the Mori–Tanaka and MOC predictions are in close alignment for all volume fraction values. It has been observed that MOCu tends to overpredict E2 in comparison to other models, particularly for fiber volume fractions greater than 0.2.

With respect to the effective shear modulus predictions presented in Figure 10b,c, it is worth noting that both the Mori–Tanaka and MOC predictions exhibit a high degree of agreement for both micromechanical models. Additionally, MOCu is identical to MOC, as the averaging process does not impact shear modulus $G_{12}$. Similarly, for $G_{23}$ (Figure 10d), the Mori–Tanaka and MOC exhibit good agreement with IM7/5320-1 for volume fractions ranging from 0.6 to 0.9. The predictions for $G_{23}$ using MOCu align with the Reuss predictions due to the MOCu 2-3 shear iso-stress condition [29].

Figure 10e,f show that the predictions for the Poisson’s ratio deviate from the traditional Voigt and Reuss bounds, indicating that these bounds are not universally applicable. In particular, the averaging procedure used in the MOC methodology has no effect on the Poisson’s ratio, $\upsilon_{12}$, yielding consistent predictions for the Mori–Tanaka, MOC, and MOCu models. While there are noticeable differences in the experimental results, all are in excellent agreement with the FEA. Conversely, the effect of MOC averaging on the Poisson’s ratio $\upsilon_{23}$ is pronounced. While the Mori–Tanaka, MOC, and MOCu models share certain predictive similarities, significant discrepancies in both values and trends are evident for all results. Importantly, these discrepancies span the entire range of volume fractions, underscoring the complex nature of Poisson’s ratio in composites.

## 6. Discussion

The experimental results presented in Figure 10 suggest that the properties of composite materials can be influenced by the fiber volume fraction. It is worth noting that none of the considered methods achieves a perfect correlation between all material properties and the fiber volume fraction parameter. This discrepancy can be attributed to various factors, such as potential inaccuracies in the measured properties of CFRPs or inherent properties of its constituents, or limitations inherent in the theoretical and FEA approaches used in micromechanics methods. While micromechanics theories provide critical insights, achieving an exact match of all layer material properties for a given fiber volume fraction remains a challenging task. In the context of isotropic constituent materials, the use of Voigt and Reuss models is a conventional approach for property prediction. However, they are known to provide a wider range of results for both longitudinal and transverse moduli compared to other micromechanical models. According to the analysis shown in Figure 10, the estimates provided by the Mori–Tanaka and Method of Cells (MOC) methods fall within an acceptable range of variance. Longitudinal evaluations and predictions of Young’s modulus typically have minimal uncertainties that apply to both materials and constituents. The micromechanics-based models of this investigation provide valuable insights for elucidating and predicting the effective properties of composite materials, which is critical for their integration into technology-driven industries.

## 7. Conclusions

This study assesses various micromechanical models for predicting the effective properties of carbon-fiber-reinforced plastic (CFRP) composites through micromechanics-based homogenization. The results are summarized as follows:

(1) An overview of commonly used micromechanics models for predicting the effective mechanical properties of composite materials is presented in this paper, outlining the foundational theories behind four analytical closed-form micromechanics models: those for the Voigt, Reuss, and Mori–Tanaka approaches as well as the MOC. The Voigt and Reuss models are simple and assume uniform stress or strain, making them less computationally intensive but less accurate for complex microstructures. The Mori–Tanaka model, on the other hand, introduces a moderate increase in complexity by incorporating interactions between inclusions and the matrix, while still remaining within a manageable computational framework. The MOC is an approach that takes into account detailed microstructural interactions and nonlinear material behavior, which can make it more computationally consuming and require more detailed modeling of the microstructure. These models serve as the theoretical basis for determining the effective mechanical properties of composite materials.
(2) This study presents a method for using finite element analysis (FEA) with a representative volume element (RVE) model to analyze computational micromechanics. The model consists of unidirectional cylindrical fibers periodically distributed within a polymer matrix, with the goal of replicating the cross-section of a unidirectional composite laminate, which is in line with established research in the field.
(3) Four analytical micromechanical models, finite element analysis, and experimental results were utilized to compare the predicted effective properties of an IM7/5320-1 unidirectional CFRP. Additionally, the variation in effective properties was examined across all micromechanics models spanning the entire range of volume fractions.

## Figures and Tables

**Table 1:** Mechanical properties of IM7 carbon fiber.

$E_{1}^{f}$ [GPa]	$E_{2}^{f}$ [GPa]	$E_{3}^{f}$ [GPa]	$\upsilon_{12}^{f}$	$\upsilon_{13}^{f}$	$\upsilon_{23}^{f}$	$G_{12}^{f}$ [GPa]	$G_{13}^{f}$ [GPa]	$G_{23}^{f}$ [GPa]
262.2	19.5	19.5	0.30	0.30	0.46	18.9	18.9	7.8

**Table 2:** Parameters of the linear Drucker–Prager model that characterize the 5320-1 epoxy matrix.

$E_{m}$ [GPa]	$\upsilon_{m}$	β	K	Ψ
3.809	0.35	31^o^	0.89	14.28^o^

**Table 3:** Material properties of IM7/CYCOM 5320-1 fiber–matrix interface.

$N_{c}$ (MPa)	$S_{c}$ (MPa)	$k_{\mathrm{nn}}^{c}=k_{\mathrm{ss}}^{c}$ (GPa/μm)	$G_{\mathrm{nn}}^{c}$ [J/m^2^]	$G_{s}^{c}$ [J/m^2^]	$\eta_{\mathrm{BK}}$
57	85	100	7	80	1.2

## Data Availability

The data are not publicly available due to the institute’s security policy.

## Appendix A. Eshelby Equivalent Inclusion Method: Dilute Dispersion Model

This appendix provides a detailed explanation of the Eshelby equivalent inclusion method for the case of a dilute distribution of inclusions within an infinite homogeneous matrix. The method is important for understanding the mechanical behavior of composite materials in which the matrix is reinforced by dispersed inclusions of different material properties. The ellipsoidal domain Ω is composed of identical material and is encased by an infinite homogeneous matrix with elastic stiffness $C^{\left(m\right)}$. This passage discusses the introduction of an eigenstrain $\varepsilon^{*}$ which was first conceptualized by Eshelby. The eigenstrain is a strain that is devoid of stress and is similar to thermal strain. It is important to note that the encasing matrix hinders the inherent shape transformation of domain Ω, resulting in internal stress. Additionally, the total strain within domain Ω is represented as ε and is the sum of eigenstrain and elastic strain. The elastic strain in the matrix material conforms to Hooke’s law, which is expressed as

(A1) $$\sigma =C^{\left(m\right)}\left(\varepsilon -\varepsilon^{*}\right)\;\mathrm{in}\;\Omega$$

According to Eshelby [42,43], if there is a uniform eigenstrain $\varepsilon^{*}$ within the ellipsoidal domain Ω, it will result in a uniform total strain ε. This validates the following linear transformation:

(A2) $$\varepsilon =P\varepsilon^{*}\;\mathrm{in}\;\Omega$$

where the components of the Eshelby tensor, denoted by P, depend on the shape of the inclusion and the material attributes of the body. The stress within the ellipsoidal domain Ω with eigenstrain $\varepsilon^{*}$ is now subject to a uniform displacement boundary condition $u_{i}\left(S\right)=\varepsilon_{ij}^{0}x_{j}$ at infinity. Given that domain Ω shares the matrix material, the stress within it is expressed as

(A3) $$\sigma =C^{\left(m\right)}\left(\varepsilon^{0}+\varepsilon -\varepsilon^{*}\right)\;\mathrm{in}\;\Omega$$

where $\sigma^{0}=C^{\left(m\right)}\varepsilon^{0}$. Consider substituting the ellipsoidal domain Ω, with stiffness tensor $C^{\left(m\right)}$, for an “equivalent” inclusion material with stiffness $C^{\left(f\right)}$, subjected to identical displacement boundary conditions and total strain ε. The stress in the domain Ω then becomes

(A4) $$\sigma^{\left(f\right)}=C^{\left(f\right)}\left(\varepsilon^{0}+\varepsilon \right)\;\mathrm{in}\;\Omega$$

The role of the eigenstrain is to ensure stress equivalency in domain Ω, whether filled by the matrix or the equivalent fiber such that $\sigma^{\left(f\right)}=\sigma$. Setting Equations (A3) and (A4) equal gives

(A5) $$C^{\left(m\right)}\left(\varepsilon^{0}+\varepsilon -\varepsilon^{*}\right)=C^{\left(f\right)}\left(\varepsilon^{0}+\varepsilon \right)$$

Equation (A4) implies that $\left(\varepsilon^{0}+\varepsilon \right)={\bar{\varepsilon }}^{\left(f\right)}$, and then it follows from Equation (A5) that

(A6) $$C^{\left(m\right)}\varepsilon^{*}=\left(C^{\left(m\right)}-C^{\left(f\right)}\right){\bar{\varepsilon }}^{\left(f\right)}$$

Combining Equations (A4) and (A2), the strain in the inclusion is

(A7) $${\bar{\varepsilon }}^{\left(f\right)}=\varepsilon^{0}+\varepsilon =\varepsilon^{0}+P\varepsilon^{*}$$

which can be reformulated as

(A8) $$\varepsilon^{*}=P^{-1}\left({\bar{\varepsilon }}^{\left(f\right)}-\varepsilon^{0}\right)$$

Substituting Equation (A8) into Equation (A6) results in

(A9) $$C^{\left(m\right)}P^{-1}\left({\bar{\varepsilon }}^{\left(f\right)}-\varepsilon^{0}\right)=\left(C^{\left(m\right)}-C^{\left(f\right)}\right){\bar{\varepsilon }}^{\left(f\right)}$$

or

(A10) $$\left[C^{\left(m\right)}P^{-1}-\left(C^{\left(m\right)}-C^{\left(f\right)}\right)\right]{\bar{\varepsilon }}^{\left(f\right)}=C^{\left(m\right)}P^{-1}\varepsilon^{0}$$

By multiplying both sides Equation (A10) by ${PC}^{(m)-1}$, one can obtain

(A11) $$\left[I-{PC}^{(m)-1}\left(C^{\left(m\right)}-C^{\left(f\right)}\right)\right]{\bar{\varepsilon }}^{\left(f\right)}=\varepsilon^{0}$$

Comparing Equation (A11) to ${\bar{\varepsilon }}^{\left(f\right)}=A_{f}\bar{\varepsilon }$, utilizing the average strain theorem, it becomes evident that the strain concentration tensor, $A_{f}$, for an ellipsoidal inclusion within an infinite matrix is

(A12) $$A_{f}^{\mathrm{dilute}\;}{\bar{\varepsilon }}^{\left(f\right)}=\varepsilon_{0}\;\mathrm{where}\;A_{f}^{\mathrm{dilute}\;}=[I-PC{]}^{-1}$$

where

(A13) $$C=C^{(m)-1}\left(C^{\left(m\right)}-C^{\left(f\right)}\right)$$

This solution for the ellipsoidal inclusion lays the groundwork for deriving various special cases of the Eshelby tensor for shapes like spheres and cylinders. Mura [71] has articulated these in tensorial notation. For example, in a spherical inclusion, the nonzero terms of the Eshelby tensor, P, in contracted notation are

(A14) $$\begin{array}{c} P_{11}=P_{22}=P_{33}=\frac{\left(7-5v_{m}\right)}{\left[15\left(1-v_{m}\right)\right]}\\ P_{12}=P_{23}=P_{31}=P_{13}=P_{21}=P_{32}=\frac{\left(5v_{m}-1\right)}{\left[15\left(1-v_{m}\right)\right]}\\ P_{44}=P_{55}=P_{66}=\frac{2\left(4-5v_{m}\right)}{\left[15\left(1-v_{m}\right)\right]} \end{array}$$

For a cylindrical inclusion that extends infinitely in the longitudinal direction within composites, the non-zero components of the Eshelby tensor P are expressed as follows

(A15) $$\begin{array}{c} P_{22}=P_{33}=\frac{\left[\frac{3}{4}+\frac{1}{2}\left(1-2v_{m}\right)\right]}{\left[2\left(1-v_{m}\right)\right]}\\ P_{12}=P_{13}=P_{21}=P_{31}=\frac{v_{m}}{\left[2\left(1-v_{m}\right)\right]}\\ P_{23}=P_{32}=\frac{\left[\frac{1}{4}-\frac{1}{2}\left(1-2v_{m}\right)\right]}{\left[2\left(1-v_{m}\right)\right]}\\ P_{44}=\frac{2\left[\frac{1}{4}+\frac{1}{2}\left(1-2v_{m}\right)\right]}{\left[2\left(1-v_{m}\right)\right]}\\ P_{55}=P_{66}=\frac{2}{4} \end{array}$$

where $v_{m}$ represents the Poisson’s ratio of the matrix. Furthermore, the factor of 2 in the contracted notation P shear components above comes from combining the two appropriate tensorial shear terms given by Mura [71], i.e., $P_{44}=P_{2323}+P_{2332},P_{55}=P_{1313}+P_{1331},P_{66}=P_{1212}+P_{1221}$.

## Appendix B. Determination of Effective Properties

In a unidirectional fiber-reinforced composite within a matrix, subjected to uniform stress or strain, the composite’s overall stiffness is represented by the following equation:

(A16) $$\sigma_{ij}=C_{ijkl}\cdot \varepsilon_{kl}$$

or

(A17) $$\varepsilon_{kl}=S_{ijkl}\cdot \sigma_{ij}$$

where $C_{ijkl}$ and $S_{ijkl}={\left[C_{ijkl}\right]}^{-1}$ are stiffness tensor and compliance tensor, respectively. For composite materials, the following engineering constants are related due to isotropy in the plane perpendicular to the fiber direction:

(A18) $$E_{22}=E_{33},\;G_{13}=G_{12},\;\nu_{12}=\nu_{13},$$

The compliance matrix of composites with transversely isotropy properties can be expressed as

(A19) $$\left[\begin{array}{c} \varepsilon_{11}\\ \varepsilon_{22}\\ \varepsilon_{33}\\ \gamma_{23}\\ \gamma_{13}\\ \gamma_{12} \end{array}\right]=\left[\begin{array}{cccccc} \frac{1}{E_{11}} & -\frac{\nu_{12}}{E_{11}} & -\frac{\nu_{12}}{E_{11}} & 0 & 0 & 0\\ -\frac{\nu_{12}}{E_{11}} & \frac{1}{E_{22}} & -\frac{\nu_{2z}}{E_{22}} & 0 & 0 & 0\\ -\frac{\nu_{12}}{E_{11}} & -\frac{\nu_{23}}{E_{22}} & \frac{1}{E_{22}} & 0 & 0 & 0\\ 0 & 0 & 0 & \frac{1}{G_{23}} & 0 & 0\\ 0 & 0 & 0 & 0 & \frac{1}{G_{12}} & 0\\ 0 & 0 & 0 & 0 & 0 & \frac{2\left(1+\nu_{12}\right)}{E_{11}} \end{array}\right]\;\left[\begin{array}{c} \sigma_{11}\\ \sigma_{22}\\ \sigma_{33}\\ \sigma_{23}\\ \sigma_{13}\\ \sigma_{12} \end{array}\right]$$

The mechanical behavior of composite materials is typically characterized by several effective mechanical properties, including the elastic modulus, $E_{11}$ and $E_{22}$ for the longitudinal and transverse directions, respectively, the effective shear modulus, $G_{12}$ and $G_{23}$, and the Poisson’s ratios, $\nu_{12}$ and $\nu_{23}$, which are fundamental for defining the mechanical behavior of composite materials. Precisely determining these properties is crucial for accurately calculating the stiffness matrix of the composite material.
